# Smart Task Assistance in Mixed Reality for Astronauts

**DOI:** 10.3390/s23094344

**Published:** 2023-04-27

**Authors:** Qingwei Sun, Wei Chen, Jiangang Chao, Wanhong Lin, Zhenying Xu, Ruizhi Cao

**Affiliations:** 1 Department of Aerospace Science and Technology, Space Engineering University, Beijing 101416, China; sunqw1992@163.com; 2 China Astronaut Research and Training Center, Beijing 100094, China; 3 National Key Laboratory of Human Factor Engineering, China Astronaut Research and Training Center, Beijing 100094, China; 4 School of Computer Science and Engineering, Beihang University, Beijing 100083, China

**Keywords:** mixed reality, astronaut training, object detection, pose estimation, point cloud alignment

## Abstract

Mixed reality (MR) registers virtual information and real objects and is an effective way to supplement astronaut training. Spatial anchors are generally used to perform virtual–real fusion in static scenes but cannot handle movable objects. To address this issue, we propose a smart task assistance method based on object detection and point cloud alignment. Specifically, both fixed and movable objects are detected automatically. In parallel, poses are estimated with no dependence on preset spatial position information. Firstly, YOLOv5s is used to detect the object and segment the point cloud of the corresponding structure, called the partial point cloud. Then, an iterative closest point (ICP) algorithm between the partial point cloud and the template point cloud is used to calculate the object’s pose and execute the virtual–real fusion. The results demonstrate that the proposed method achieves automatic pose estimation for both fixed and movable objects without background information and preset spatial anchors. Most volunteers reported that our approach was practical, and it thus expands the application of astronaut training.

## 1. Introduction

Traditional astronaut training methods contain instructor-led instruction, querying manuals, voice guidance, etc. However, these methods require astronauts to memorize and query operational processes, increasing the task burden [1,2]. To solve this problem, MR is applied to provide virtual assistance information, such as process guidance [3], equipment operation simulation [1], etc., Refs. [4,5], on the astronaut’s headset. In practice, MR provides astronauts with intuitive operation guidance, freeing them from complicated manuals, reducing brain load and operation errors, and meeting the needs of autonomous training.

The core of MR is the fusion of virtual information and real objects in 3D space. The key point is to determine the pose of objects relative to the MR device to ensure that the hologram can be accurately projected. In application development, spatial anchors are generally preset [6], and the pose of the virtual information is adjusted manually. However, this mode is only applied in scenes with fixed objects due to the lack of automatic pose estimation. As the environment changes significantly, spatial anchors need to be updated. Some presented works also propose preload markers on the object, which can solve the problem effectively [7,8]. However, we do not want to add extra information to the natural scene when training. Neither of these models can meet the application requirements for moving objects in the environment. 

For the reasons above, conventional MR devices only perform recognition of the geometric space and cannot detect specific objects or perceive the dynamic changes in the scene. To meet the MR training needs, it is crucial to develop a method that is not limited by static space.

We break down this issue into discovering objects and estimating poses. Deep learning-based object detection is widely used to detect objects. According to whether the method generates proposals, object detection is divided into two types, namely two-stage methods [9,10,11,12,13] and one-stage methods [14,15,16,17,18,19]. Two-stage methods are characterized by generating proposals first and then classifying the objects and are slower than one-stage methods. 

The purpose of pose estimation is to determine the 6DoF pose of the object in 3D space, including translation and rotation. Generally, there are three types of methods to solve this problem. The first is the correspondence point-based method [20,21,22,23,24], which finds the matching relationship between the input data and the point cloud of the object. The second is the template-based approach [25,26,27,28], where a template similar to the object is selected from a marked pose library. The third is the voting-based approach [29,30,31], with the idea that each 2D pixel or 3D point contributes to the 6DoF pose. In MR applications, the low-resolution images acquired by visual sensors and the soft texture of the astronaut training environment prevent the stable extraction of 2D features. Fortunately, TOF or stereo cameras can obtain the object’s depth, so the ICP algorithm can be used.

In work similar to ours [32], Mask-RCNN is used to segment instances. Both 2.5D and 3D replicas are extracted from the spatial mapping generated by instances to enhance the perception of the scene. Furthermore, Mask-RCNN is also used in the subsequent work [33], and the CAD point cloud is aligned with the object point cloud through the ICP algorithm. Unlike our method, Park et al.’s work requires the virtual model to be registered to markers beforehand. At the same time, the background of the astronaut training environment is more complex, and the object is quite small, making pose estimation challenging.

To address the limitation that MR cannot automatically detect objects, we propose a pose estimation method based on object detection and point cloud alignment. Indeed, objects in astronaut training are small. They occupy a small percentage of pixels, which poses a significant challenge to traditional image processing methods. In practice, we use YOLOv5s [14] to detect small samples and irregular objects. Furthermore, the partial point cloud is segmented and is ICP-aligned [34] with the template point cloud to estimate the pose of objects. 

In experiments, fixed and movable objects are selected for MR. Subjects with different backgrounds are invited to experience our system. Results show that the proposed method effectively improves MR scene comprehension and extends the methods of astronaut MR training. The contributions of this study are as follows:

1. We propose a smart MR task assistance method based on object detection and point cloud alignment to meet the requirements of pose estimation of fixed and movable objects.
2. Aiming at the specific setting of astronaut training, YOLOv5s and ICP are used to detect objects and calculate poses, respectively.
3. The results demonstrate the usability and usefulness of the proposed method, providing a new method for subsequent research.

## 2. Materials and Methods

Our method focuses on object detection and pose estimation in astronaut training and guides the virtual information to be accurately rendered onto the real object. Specifically, we remove the limitation of preset poses and solve the problem of inaccurate registration between virtual information and movable objects. Unlike in the work of Park et al. [33], the objects in our images are small, and the depth measurement is inaccurate. Therefore, the point cloud obtained from a single view is incomplete, which poses a significant challenge to the pose estimation. Given this scene’s characteristics, YOLOv5s is combined with the ICP algorithm to execute pose estimation in MR applications. Firstly, YOLOv5s is used to find the object’s bounding box on the RGB image. Secondly, the partial point cloud of the object is calculated based on its corresponding depth value. Finally, the object’s pose is calculated by aligning it with the template point cloud. Furthermore, the pose is applied to the astronaut MR to enhance the system’s robustness and smartness. 

### 2.1. Overview of the Proposed Method

**Figure 1:**
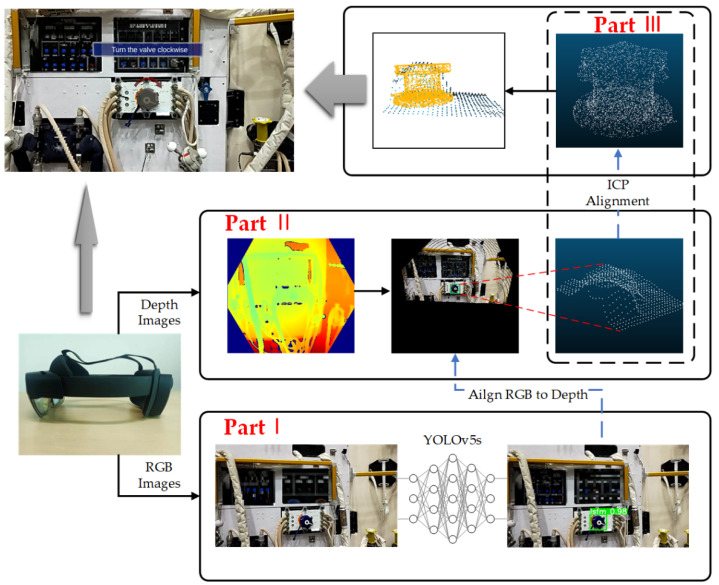
Structure of the proposed method. The method is divided into object detection, generation of the partial point cloud, and ICP.

As shown in Figure 1, the algorithm utilizes HoloLens2 as the MR device and is divided into three main parts. Part I is object detection, where YOLOv5s is used to obtain the object’s bounding box on the RGR image, i.e., the pixel coordinates of the four corners of the rectangular box. Part II is the generation of the partial point cloud, including (a) alignment of the RGB image with the depth image, (b) determination of the coordinates of the bounding box on the depth image, and (c) calculation of the point cloud of the corresponding region based on intrinsic parameters and depth values. Part III calculates the pose of the partial point cloud relative to the template point cloud, i.e., the object’s pose relative to the camera coordinate system, using ICP, where the template point cloud is obtained by sampling from the CAD model.

The world coordinate system of the camera can be obtained through SLAM and the extrinsic parameters between sensors in HoloLens2. Therefore, the object’s pose relative to the world coordinate system, i.e., a fixed position in 3D space, can be obtained. Furthermore, this pose can register the virtual information used for astronaut training with the real object.

Communication between HoloLens2 and the server is performed via UDP. HoloLens2 transmits depth, RGB images, and each sensor’s intrinsic and extrinsic parameters to the server. The server sends the object’s pose to HoloLens2. The pose includes translation and rotation, where the translation is defined as t=[x,y,z], and rotation is defined as a quaternion: q=[a,b,c,w]. Considering the performance of HoloLens2 and the server’s processing capacity, the data-sending frequency of HoloLens2 is three fps. If the frequency is too high, it will store the data in the queue and increase the latency. Indeed, the latency becomes more pronounced as the program runs longer. In practice, the time consumption of ICP on our device is about 0.187 s per frame, which will be completed before the subsequent frame transmission.

### 2.2. Transformation of Coordinate Systems

**Figure 2:**
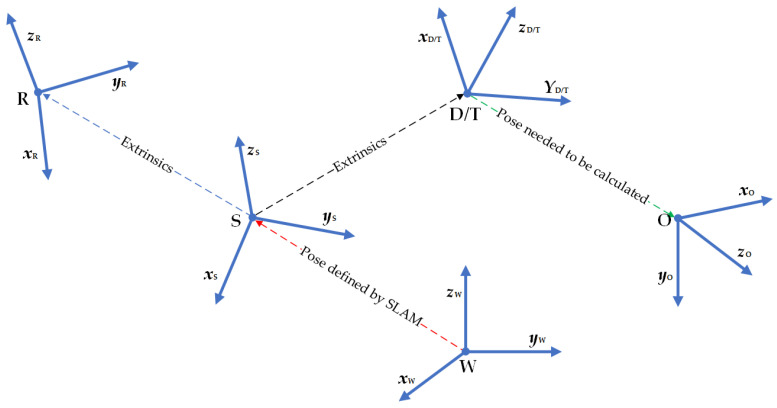
Coordinate systems used in the method.

The proposed method involves transformation between multiple coordinate systems, as shown in Figure 2. W is the world coordinate system, the initial coordinate system of the entire system, determined by the HoloLens2 boot position. S is the system coordinate system of HoloLens2, called the *rigNode* in [35], and HoloLens2 provides extrinsic parameters of all sensors relative to S. R is the RGB camera coordinate system. D is the depth camera coordinate system. We align the RGB image to the depth image.

From the transformation $T_{\mathrm{SR}}$ of R relative to S and the transformation $T_{\mathrm{SD}}$ of D relative to S, the following relationship can be obtained:

(1) $$T_{\mathrm{DR}}=T_{\mathrm{DS}}\times T_{\mathrm{SR}}=T_{\mathrm{SD}}^{-1}\times T_{\mathrm{SR}}.$$

T is the coordinate system where the template point cloud is located. In our method, T is set to be the same as D to simplify the pose calculation’s complexity. O is the coordinate system where the object is located.

The key to MR is to determine the transformation of O relative to S, i.e., to find $T_{\mathrm{SO}}$, which is expressed as a translation $t_{\mathrm{SO}}$ and a rotation $R_{\mathrm{SO}}$. $T_{\mathrm{WS}}$ can be obtained by SLAM, which is considered known. Therefore, by obtaining $T_{\mathrm{SO}}$, the absolute coordinates of the object in the world coordinate system can be defined as $T_{\mathrm{WO}}=T_{\mathrm{WS}}\times T_{\mathrm{SO}}$. T coincides with D, so define $T_{\mathrm{SD}}=T_{\mathrm{ST}}$, which can be obtained from extrinsic parameters and is considered known. The process of calculating $T_{\mathrm{SO}}$ can be translated into calculating $T_{\mathrm{SO}}=T_{\mathrm{SD}}\times T_{\mathrm{DO}}$, i.e., $T_{\mathrm{SO}}=T_{\mathrm{ST}}\times T_{\mathrm{TO}}$. Our goal is to calculate $T_{\mathrm{TO}}$.

Note that the above coordinate system follows a right-handed coordinate system. The application is developed using Unity, which follows a left-handed coordinate system, so the pose obtained by HoloLens2 needs to be transformed into a left-handed coordinate system. In the coordinate system transformation, a distinction should be made between translation and rotation, and the two cases should be handled separately [36].

The translation involves a transformation of the point positions as long as the corresponding axes are inverted. Taking the Z-axis as an example, the point $P_{r}\left(x,y,z\right)$ in the right-hand system is transformed into the point $P_{l}\left(x,y,-z\right)$ in the left-hand system, which is represented by the matrix as the following:

(2) $$P_{l}=\left[\begin{array}{c} x\\ y\\ -z \end{array}\right]=\left[\begin{array}{ccc} 1 & 0 & 0\\ 0 & 1 & 0\\ 0 & 0 & -1 \end{array}\right]\left[\begin{array}{c} x\\ y\\ z \end{array}\right]=S_{t}P_{r}.$$

The transformation matrix for translation is as follows:

(3) $$S_{t}=\left[\begin{array}{ccc} 1 & 0 & 0\\ 0 & 1 & 0\\ 0 & 0 & -1 \end{array}\right].$$

Assuming that the rotation matrix in the right-handed system is $R_{r}$, and the rotation matrix in the left-handed system is $R_{l}$, then we define the following:

(4) $$R_{l}=S_{t}R_{r}S_{t}.$$

Equation (4) is the rotation matrix in the left-handed coordinate system. Equations (2) and (4) can be used to convert the poses in the right-handed coordinate system over to the left-handed coordinate system.

### 2.3. Object Detection Based on YOLOv5s

#### 2.3.1. YOLOv5s

There are several versions of YOLOv5, among which YOLOv5s is the simplest. The remaining ones are extended on this basis. Astronaut training scenes are relatively unchanged and do not require the network to have too high of a generalization ability, so that YOLOv5s can meet the demand.

YOLOv5s is divided into four parts: input, backbone, neck, and prediction. Among them, the input adopts Mosaic data enhancement. In practice, four images are stitched together by random scaling, random cropping, and random arrangement, which improves the detection of the small object. This reduces training dependence on batch size and is more suitable for the particular scene of astronauts. Moreover, Focus and CSP (Cross Stage Partial Network) are used in the backbone. The Focus slices the image, expands the input image of three channels to twelve channels, performs sampling of images, and retains information completely, compared with traditional sampling. The CSP performs a two-step operation on feature maps: one is convolution, and the other combines the results of the previous convolution, similar to ResNet [37]. CSP brings significant improvement, effectively enhancing the ability of CNN and reducing calculation. Then, the neck part changes the convolution to the CSP, further improving the network’s power. In the prediction part, YOLOv5s fuses the results of three different resolutions and maps them to the same size as the input image.

The above characteristics make the YOLOv5s able to be trained faster with small datasets and detect small-sized objects more accurately. So, we choose YOLOv5s as the network for object detection.

#### 2.3.2. Datasets

**Figure 3:**
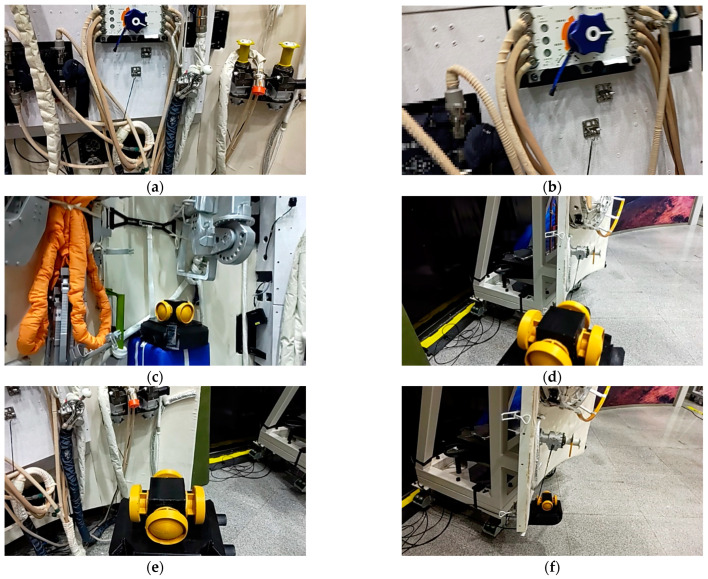
Some images used in our method. (**a**,**b**) are fixed objects; (**c**) is the movable object in scene 1; (**d**,**e**) are objects during movement; (**f**) is the movable object in scene 2.

Astronauts work in a relatively unchanged environment, and two frequently operated objects are selected, including a valve and a panoramic camera. We use HoloLens2 to scan the scene, and the data stream is saved as images. Five hundred are selected, 400 of which are used as the training set and the other 100 as the validation set. Some of the data are shown in Figure 3.

It should be noted that some of the data are blurred, as shown in Figure 3. 

Figure 3b is normal and reasonable. This is caused by the movement of the HoloLens2 while capturing images, which often happens in natural application scenes.

A large amount of labeled data like COCO cannot be acquired in the experiment, and the image resolution captured by HoloLens2 is poor. Compared with the general case, this application is characterized by small samples and low resolution, which creates higher requirements. Fortunately, the astronaut training scene is unchanged. For object detection, there is a high similarity between the train and test datasets, so the strong generalization ability of the network is not required. Meanwhile, Mosaic and self-adversarial trainings (SAT) are used in YOLOv5s for data augmentation, which expands the dataset.

#### 2.3.3. Network Training

The developing environment and parameters are shown in Table 1, and YOLOv5s is fine-tuned based on the pre-training weights. Since our dataset is relatively small, we use a small batch size to meet the training requirements. The optimization algorithm is stochastic gradient descent (SGD), and CUDA accelerates the training.

**Figure 4:**
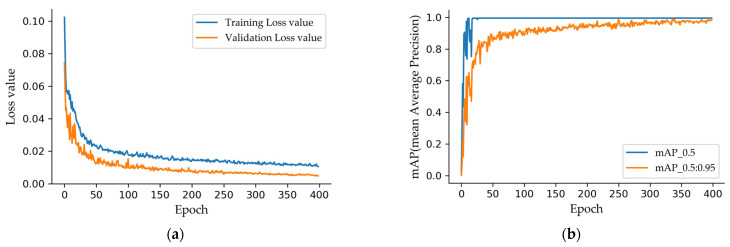
Training results. (**a**) Loss of training and validation; (**b**) mAP of validation dataset.

Figure 4a shows the training loss curves, which decrease rapidly in the first 50 epochs and stabilize after 200 epochs. The mAP (mean Average Precision) of the validation set is shown in Figure 4b. When the IoU (Intersection over Union) threshold is set to 0.5, mAP stabilizes at a high value (fixed at 0.995) after 30 epochs. When the threshold is set from 0.5 to 0.95, the average mAP is stabilized at a high value after 300 epochs. The proposed method uses the best weights after 300 epochs for object detection. It should be noted that the training is easy because the network is not trained from scratch, and the dataset is small compared to COCO. 

### 2.4. Generation of Partial Point Cloud

As shown in Part II in Figure 1, the method is divided into object detection, generation of the partial point cloud, and ICP.

The RGB image captured by HoloLens2 is used to determine the object’s bounding box by YOLOv5s, represented by four values (*x*, *y*, *w*, *h*). (*x*, *y*) is the center coordinate of the bounding box, and (*w*, *h*) is the width and height of the bounding box.

The resolution of the RGB image acquired by HoloLens2 is 726×428, and the resolution of the depth image is 320×288. The field of view of the two datasets differs significantly, so the two images need to be aligned. The point cloud corresponding to the depth image can be obtained according to intrinsic parameters. Assume that the coordinate of the point $P_{D}$ in the depth coordinate system is ${\left[X_{D},Y_{D},Z_{D}\right]}^{T}$, which is calculated as

(5) $$P_{D}=\left[\begin{array}{c} X_{D}\\ Y_{D}\\ Z_{D} \end{array}\right]=Z_{D}K_{D}^{-1}\left[\begin{array}{c} u_{D}\\ v_{D}\\ 1 \end{array}\right],$$

where ${\left(u_{D},v_{D}\right)}^{T}$ is the pixel coordinate corresponding to the point in the depth image, $Z_{D}$ is the depth of $P_{D}$, and $K_{D}$ represents the intrinsic parameter of the depth camera. From the transformation $T_{\mathrm{DR}}$ of the RGB camera coordinate system relative to the depth camera coordinate system, the spatial point coordinates can be calculated as

(6) $$P_{R}=T_{\mathrm{RD}}P_{D}=T_{\mathrm{DR}}^{-1}P_{D},$$

where $T_{\mathrm{DR}}$ is the transformation of the depth camera coordinate system relative to the RGB camera coordinate system. Since $Z_{D}\approx Z_{R}$, the pixel coordinates ${\left(u_{R},v_{R}\right)}^{T}$ on the RGB image can be calculated as follows:

(7) $$\left[\begin{array}{c} u_{R}\\ v_{R}\\ 1 \end{array}\right]=Z_{R}^{-1}K_{R}P_{R}=Z_{R}^{-1}K_{R}T_{\mathrm{DR}}^{-1}P_{D},$$

where $Z_{R}$ is the depth of $P_{D}$ in the RGB camera coordinate system. By combining Equations (5) and (7), it can be calculated as follows:

(8) $$\left[\begin{array}{c} u_{R}\\ v_{R}\\ 1 \end{array}\right]=K_{R}T_{\mathrm{DR}}^{-1}K_{D}^{-1}\left[\begin{array}{c} u_{D}\\ v_{D}\\ 1 \end{array}\right].$$

**Figure 5:**
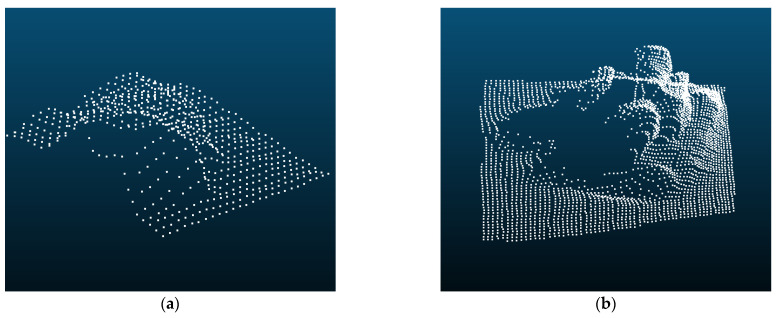
Partial point cloud captured by HoloLens2. (**a**) Partial point cloud of the valve; (**b**) Partial point cloud of the panoramic camera.

In practice, we align RGB images to depth images through Equation (8). Then, corresponding pixel coordinates of the bounding box on the depth image can be determined. Furthermore, we obtain the point cloud according to Equation (5). It should be noted that the point cloud is generated by the single-view depth image, which only expresses partial information about the object and is called the partial point cloud. As shown in Figure 5, the point cloud is incomplete where the camera cannot capture the whole structure due to obstruction.

### 2.5. Pose Estimation

**Figure 6:**
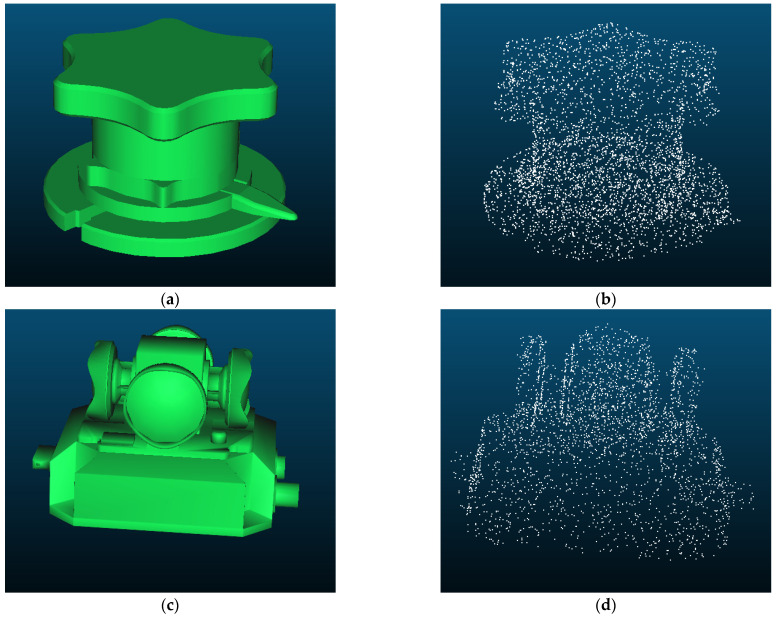
Template point clouds in this method. (**a**) CAD model of a valve; (**b**) The template point cloud obtained by sampling the CAD model of the valve; (**c**) The CAD model of a panoramic camera; (**d**) The template point cloud obtained by sampling the CAD model of the panoramic camera.

The template point cloud is obtained from the manually created CAD model by sampling, as shown in Figure 6. The partial point cloud is aligned with the template point cloud by ICP to calculate the transformation between them. The commonly used ICP algorithms are divided into the point-point algorithm [34] and the point-plane algorithm [38], and the point-plane algorithm converges faster. However, it is found in the experiments that the results calculated by the point-plane algorithm often fail. The reason may be that point clouds in this method are too noisy and non-homologous. Additional errors are introduced in determining the plane where the points are located, which is not as good as directly calculating the distance between matched pairs of points. The ICP method used in this method is as follows.

Given point clouds $P=\left(p_{1},p_{2},\ldots ,p_{m}\right)$ and $Q=\left(q_{1},q_{2},\ldots ,q_{n}\right)$, we define the target function:

(9) $$E\left(T\right)=\sum_{\left(p,q\right)\epsilon \Omega }\rho \|p-Tq\|,$$

equivalent to

(10) $$E\left(T\right)=\underset{\left(p,q\right)\epsilon \Omega }{\sum }\rho \|p-Rq-t\|.$$

Among them,

(11) $$\rho \left(x\right)=\frac{\mu x^{2}}{\mu +x^{2}}$$

*P* is the template point cloud, and *Q* means the partial point cloud. *T* denotes the transformation matrix between point clouds, including the rotation *R* and the translation *t*. ρ is the kernel function to limit the influence of outer points. Then, the pose between point clouds can be calculated by minimizing Equation (10). In practice, fast global registration (FGR) [39] is used to obtain the initial transformation.

**Figure 7:**
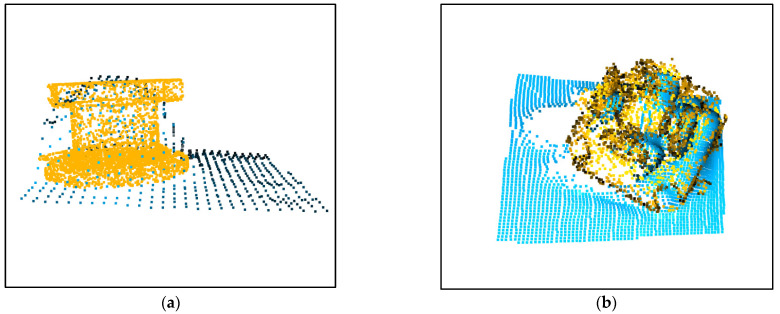
Results of the point cloud alignment. (**a**) Results of the valve; (**b**) Results of the panoramic camera.

The results of the point cloud alignment are shown in Figure 7. Although the appearance of the two point clouds is different, the alignments are approximately accurate.

## 3. Results and Discussion

A fixed valve and a movable panoramic camera are selected as detected objects to verify the method’s performance. Given that the MR ultimately provides operational guidance to humans, human experience in the task is essential. Twenty subjects are invited to experience HoloLens2 and give feedback on whether the algorithm is useful through questionnaires. The experiments show that the proposed method can project virtual information with fixed or movable objects, proving the usability and usefulness of the method.

### 3.1. Results of Object Detection

After 100 images were tested, the average processing time for each image was 0.0102 s, including 0.0005 s for preprocessing, 0.0082 s for inference, and 0.0015 s for NMS (non-maximum suppression). Compared with the data transfer rate between HoloLens2 and the server, the object detection time is negligible, so the operation of the whole system can be well satisfied.

**Figure 8:**
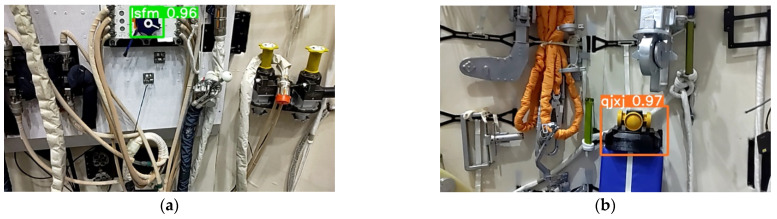

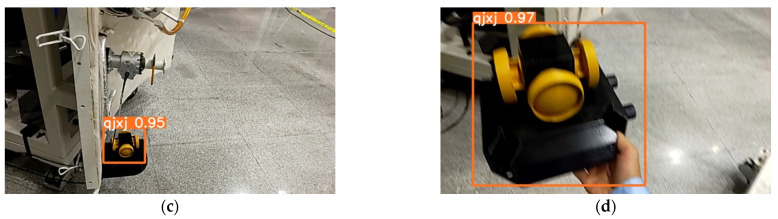
Results using YOLOv5s. (**a**) Result of the valve (the label is set to *lsfm*); (**b**,**c**) Results of the fixed panoramic camera (the label is set to *qjxj*); (**d**) Result of the moving panoramic camera.

Compared to work dedicated to object detection, it does not make sense for us to verify the accuracy of the network on a test dataset. This is because the test set of our application scene is similar to the training set, which is evident in the training phase of the network. The detection results saved during the whole system’s real-time operation are shown in Figure 8. Figure 8a is the result of a fixed valve, Figure 8b,c are results of a fixed panoramic camera, and Figure 8d is the result of a moving panoramic camera. It can be seen from Figure 8d that the network accurately detects the object even if the captured image is blurred while on the move. The images sent from HoloLens2 to the server were all detected correctly.

### 3.2. MR Applications for the Static Object

The experimental object is a valve fixed to the cabin. In astronaut training, it is usually necessary to operate similar objects. The astronauts could be guided to perform the correct operation with added virtual information. This is particularly important in space–ground communication, where the information had by astronauts in orbit and experts on the ground is not equivalent. Astronauts in space will be less sensitive to orientation for various unique reasons, so MR assistance is especially needed.

**Figure 9:**
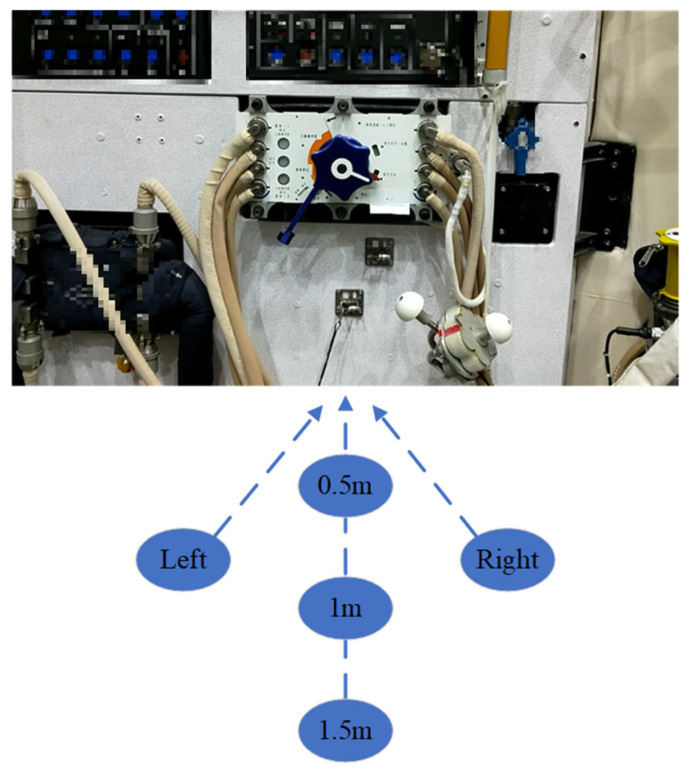
Observe the object from different distances and angles.

The marker-based approach is more sensitive to distance [32]. For comparison, a valve is observed at different distances and angles to verify the proposed method, as shown in Figure 9. Due to the spacecraft cabin structure limitation, the experiment performs the observation at distances of 0.5 m, 1 m, and 1.5 m, respectively. Furthermore, we change the observation angle at the fixed distance of 1 m.

**Figure 10:**
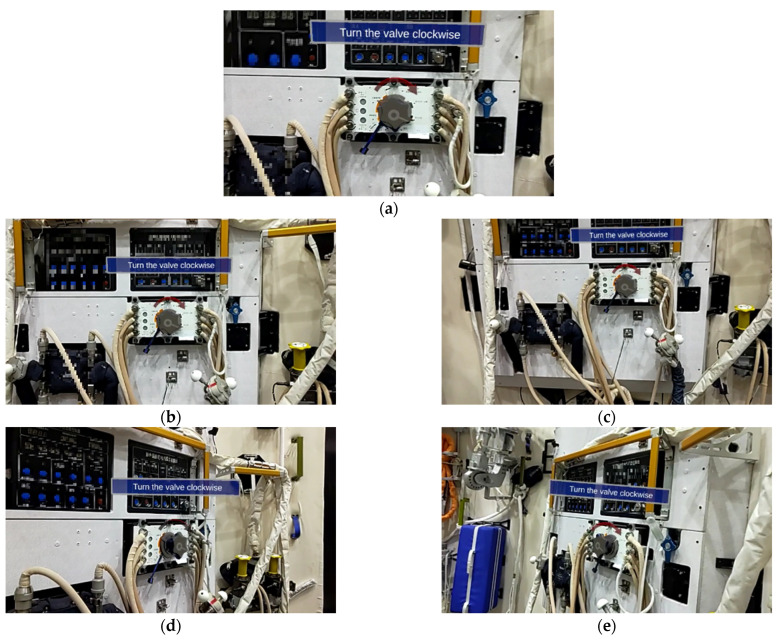
Results from different distances and angles of our method. (**a**) 0.5 m; (**b**) 1 m; (**c**) 1.5 m; (**d**) Left; (**e**) Right.

Figure 10 shows the results of our algorithm at each point. It can be seen that the virtual–real fusion is good when facing the object regardless of the distance change. However, it must be noted that the virtual–real matching worsens when the observation angle changes. Because the object’s front surface is contoured, the observation is complete. In comparison, the brim is incomplete when the occlusion occurs from the rotation. Astronauts generally face the object during operation, so the inaccuracy of the lateral pose will not affect the task result.

**Figure 11:**
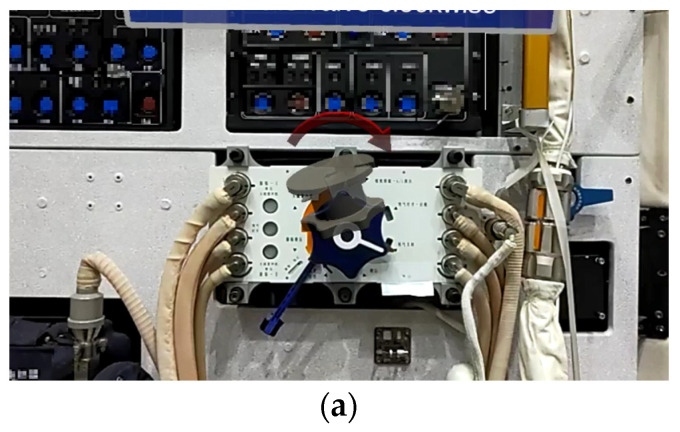

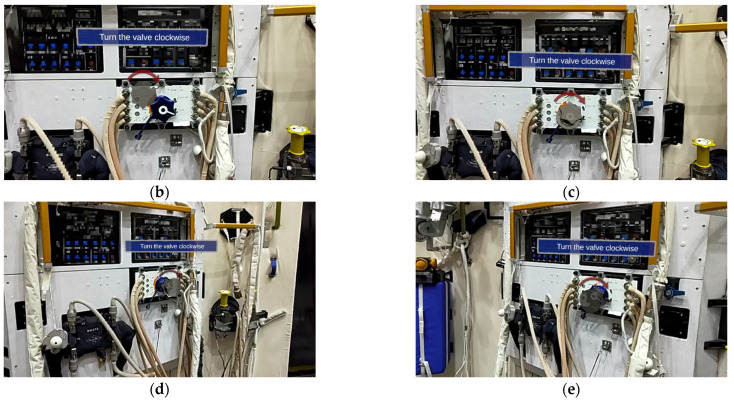
Results from different distances and angles of [33]. (**a**) 0.5 m; (**b**) 1 m; (**c**) 1.5 m; (**d**) Left; (**e**) Right.

The most similar method to ours is [33]. Figure 11 shows the results using the algorithm proposed in [33]. To be clear, our results are all screenshots of HoloLens 2 running in real-time. Therefore, they do not coincide with the viewing angles in Figure 10. As can be seen, it is hard to get accurate results. For [28], the subjects were large-sized objects with a simple background. By contrast, our object size is small, and the environment is more complex. So, our method is more suitable for astronaut training.

**Figure 12:**
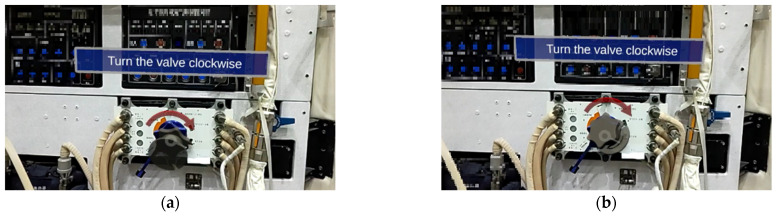
Some of the poor registration results. (**a**,**b**) are two poor registration results.

In particular, during the experiment, the virtual-real registration is ineffective in some cases. As shown in Figure 12, the axis of symmetry of the virtual valve differs from that of the real object. The cause is the different depth data from different bounding boxes, which results in the difference in ICP. To solve this problem, two methods are proposed.

#### 3.2.1. Filtering Results of the Pose

**Figure 13:**
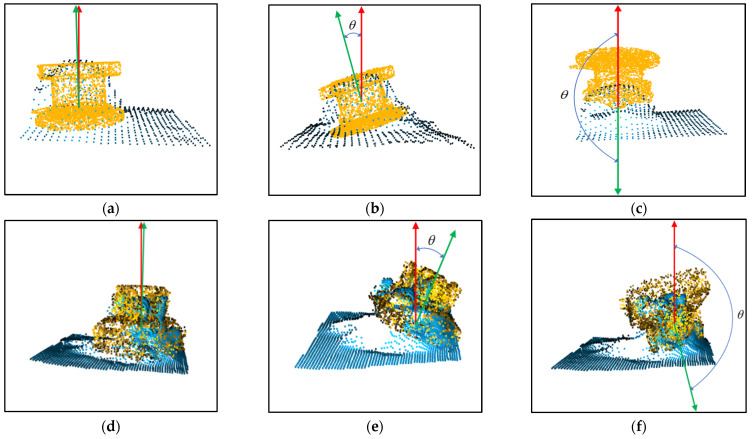
Different results in the ICP. (**a**–**c**) Results of the valve; (**d**–**f**) Results of the panoramic camera.

The pose is sent to HoloLens2 only when the angle θ between the symmetry axis of the hologram and the symmetry axis of the real object does not exceed the threshold. The previous pose is always used until the new pose is received, and the threshold is set to 5°. Therefore, helpful results will always be displayed during the device’s operation. Various results are shown in Figure 13. The angle between the symmetry axis of the template point cloud (green) and the symmetry axis of the partial point cloud (red) is θ. In practice, the results with θ less than the threshold are selected and sent to HoloLens2 for display.

#### 3.2.2. Only the Translation Is Calculated

**Figure 14:**
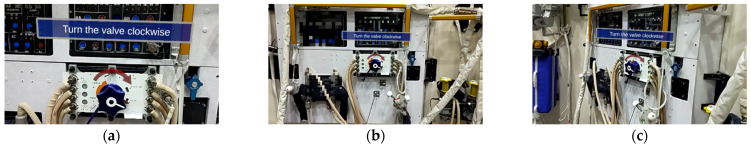
Only the translation is calculated, and the virtual valve is hidden. (**a**–**c**) are results of different orientations.

During the ICP, we find that the translation is calculated robustly. However, the rotation error is relatively large. The virtual arrow (red) and text in Figure 14 are artificially set to rotate with the line of sight (always directly in the line of sight) and are fixed relative to the valve. Therefore, it is necessary to know the translation of the valve only to determine the position of the virtual arrow and the text, not to precisely calculate the rotation of the valve.

Figure 14 shows the results of using this method and hiding the valve. There may be errors in the rotation of the virtual valve calculated by ICP. While the translation is more accurate, the red arrows and the text can always be displayed correctly without rotation. Astronaut training primarily includes process tasks. Specifically, tips on the operation are required, but not frequent registration of real and virtual objects. Therefore, our method can meet the task needs.

### 3.3. MR Applications for the Movable Object

**Figure 15:**
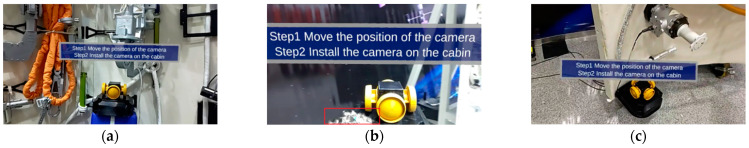
Results for moving targets. (**a**) Scene 1; (**b**) During the movement (in the red rectangle is the hand recognition by HoloLens2); (**c**) Scene 2.

Moving positions and performing operations on objects are necessary for some training, such as assembling equipment. A typical task is moving the panoramic camera in astronaut extravehicular activities. Conventional MR only performs localization by the marker or background and does not work when the object moves to a new location. To address this issue, a panoramic camera is used in the experiment. Figure 15a shows the result of virtual-real registration in scene 1, and the text is accurately displayed above the panoramic camera. The panoramic camera is moved to different scenes, and Figure 15b shows the moving process. As shown in Figure 15c, when the panoramic camera appears again in a different field of view, the virtual information can be quickly matched on the real object. Experiments show that the proposed method does not depend on any background and does not require any fixed reference to be preset. The registration can be performed as long as the object is detected in the field of view.

### 3.4. Feedback from Subjects

MR is ultimately used to assist astronauts, so the human experience is significant. Fifteen subjects (seven men and eight women, with an average age of 30.2 years) are invited to the experiment. Among them, five have MR development experience, another five have no development experience but do have experience in MR applications, and the other five do not have access to MR equipment. Subjects are asked to wear HoloLens2 and are allowed to move around to observe the fixed object. Then, the panoramic camera can be carried to other sites at will so the subject can experience the method’s performance with a movable object. After the experiment, each subject must finish a questionnaire consisting of 10 questions, as shown in Table 2, several of which are acquired from [33]. Each question has a score of 10. A higher score indicates that the proposed method is more effective, and the average score for each question is calculated to assess the method’s performance.

**Figure 16:**
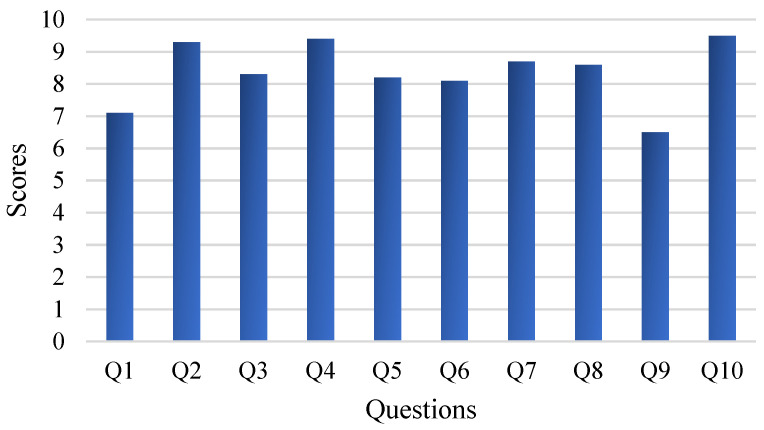
Results of the questionnaires. Higher scores indicate that the proposed method is more useful.

Figure 16 shows the results of the questionnaires (averaged). There is a consensus that this method is better when applied to movable objects, especially for those with experience in MR development. Because the traditional method generally applies to static environments, where markers or spatial anchors are preset in advance, it cannot be used for movable objects. While in the static task, subjects who do not have access to MR have a positive experience, other subjects do not see much improvement over the traditional method. Results are sometimes inferior to the traditional method in terms of pose accuracy. This is because the traditional method can manually preset the virtual object and precisely adjust the poses according to human observation.

In contrast, achieving precise results in our method is difficult due to the quality of the point cloud and the alignment error. The same issue is reflected in Q5 and Q6, where some subjects encountered poor pose accuracy during the experiment. Indeed, the pose estimation is performed in real-time. The quality of the partial point clouds obtained at different angles will lead to different pose accuracy. Although two methods are adopted to correct this problem, the accuracy is still inadequate compared to markers or spatial anchors. 

Some subjects feel that precise alignment of the virtual object with the real object is not very helpful for the task and that only text is needed to satisfy the requirements, as reflected in Q3 and Q4. This is because the task focuses on affecting the operational process and does not require precise guidance to perform delicate operations on the object. At the same time, the proposed method makes it difficult to precisely match the virtual object with the real object. The text, on the other hand, only needs to calculate the translation of the real object, so more subjects prefer to use the procedure without the virtual object. Q7 and Q8 are mainly reflected in the setting of virtual information, which has little relationship to pose accuracy. Our method adds text for information enhancement, which is more robust with pose accuracy. The LAN limits the data transmission rate to 3 fps, so there is a delay when the object is moved to a new location. Therefore, some subjects feel that the real-time performance is unsatisfactory, which is reflected in Q9.

In summary, most subjects approve of our system and consider it a meaningful attempt. There is still room for improvement, especially regarding pose accuracy. Of course, this needs to be accomplished through other methods. Methods based on deep learning and point cloud alignment are inherently uncertain, and it is challenging to achieve the same accuracy with them as it is with manual fine-tuning.

## 4. Conclusions

A method based on object detection and point cloud alignment is proposed for task assistance in astronaut MR training. For the weak texture features, small object size, and irregular configuration, YOLOv5s is used to detect the object’s bounding box on RGB images. Furthermore, depth images are combined to segment the object’s partial point cloud. Then, the partial point cloud is aligned with the template point cloud using ICP, where the template point cloud is obtained by sampling from the CAD model. The proposed method does not rely on any pre-determined spatial information. It automatically estimates the pose of fixed or movable objects, expanding the forms of MR training for astronauts.

Experiments are performed for fixed and movable objects, respectively. Specifically, virtual information can be accurately projected onto fixed objects and does not depend on anchors. For the movable object, the virtual information can follow the object, which improves the flexibility of MR training. Moreover, the subject experience shows that the proposed method has better value for movable objects. However, the accuracy of virtual-real alignment is not improved. For future work, different technologies or devices need to be used to enable higher pose accuracy and faster data transmission. 

## Figures and Tables

**Table 1:** The learning environment for YOLOv5s.

Environment	NVIDIA GeForce GTX 2060, Pytorch GPU, Intel i7-8750H
Network	YOLOv5s
Additional dataset	400 images for each additional class
Pre-train dataset	COCO
Epochs	400
Learning rate	0.0001
Batch size	4

**Table 2:** Questionnaires for the proposed method.

	Simplified Questions
Q1	Like using this system on the fixed object
Q2	Like using this system on the movable object
Q3	Like using this system with virtual models and text
Q4	Like using this system without virtual models
Q5	Projected virtual information on the real object naturally
Q6	Effective registration of real structure and virtual information
Q7	Helpful for understanding structure operations
Q8	It is easy to understand the meaning of virtual information
Q9	The real-time performance of the method is acceptable
Q10	It will be an effective method

## Data Availability

Due to the policy of the affiliation, data sharing is not applicable to this article.
